# Adaptive Management to Reduce Nest Inundation of a Critically Endangered Freshwater Turtle: Confirming the Win-win

**DOI:** 10.1007/s00267-022-01601-2

**Published:** 2022-02-07

**Authors:** Tom Espinoza, Sharon M. Marshall, Duncan J. Limpus, Col J. Limpus, Andrew J. McDougall

**Affiliations:** 1 Department of Regional Development, Manufacturing and Water, Bundaberg, QLD 4670 Australia; 2 Department of Environment and Science, Dutton Park, QLD 4102 Australia

**Keywords:** Sustainable development, Water storage management, Elseya, White-throated snapping turtle, Environmental flows

## Abstract

Inundation of Australian freshwater turtle nests has been identified as a threat to recruitment and long-term viability of species such as the critically endangered white-throated snapping turtle (Elseya albagula). Water level fluctuations within water storage infrastructure can inundate significant proportions of E. albagula nests in any year. Using an ecological risk assessment framework, operating rules for a water storage in the Burnett River (South East Queensland, Australia) were implemented to support nesting of E. albagula. Turtles were encouraged to nest at higher elevations on riverbanks by maintaining higher water levels in the impoundment during the nesting season, followed by lowering of water levels during the incubation period to minimise rates of nest inundation from riverine inflows. To verify the success of the new rules, a three-year confirmation monitoring program of nest heights and water levels was undertaken. Results of confirmation monitoring showed that 3% (2018), 11% (2019) and 0% (2020) of E. albagula nests were inundated under the new operating rules, compared to previously estimated nest inundation rates of >20% in ~24% of years of a 118-year simulation period (1890–2008) under previous storage operating rules. Emergency releases from an upstream storage in 2019 and 2020 for dam safety did not affect the success of the rule, demonstrating its resilience to natural and artificial flow regimes. This study demonstrates the importance of confirmation monitoring in verifying the efficacy of targeted changes to water management, and highlights potential application across other water storage infrastructure with threatened freshwater turtle populations requiring adaptive management.

## Introduction

Despite numerous warnings of extinction risks, and calls for more comprehensive ecological information, turtles remain one of the most endangered and poorly understood vertebrate taxa in the animal world (Lovich et al. 2018; Macip-Ríos et al. 2015; Stanford et al. 2020). For freshwater turtles in Australia, predicted declines in abundance are now being confirmed quantitatively (Chessman 2011; Van Dyke et al. 2019). Declines in ecosystem condition and resilience coupled with increased recognition of the ecosystem services provided by turtles; are driving efforts to improve resource management for this guild of freshwater species (Santori et al. 2020; Sinha 1995). Human impacts on freshwater turtles however, affect all life-stages, and complexities within their own life-histories means multiple management strategies are required to meet this challenge (Spencer et al. 2017).

Freshwater turtles alternate between aquatic and terrestrial environments throughout their life-history. Even before an egg has hatched, freshwater turtles are subject to multiple human impacts, both direct and indirect. If a female turtle has managed to reach maturity, find a mating partner, and access a suitable riverbank with the required environmental conditions for nesting; the egg and nest must still avoid being harvested, predated, trampled, inundated or dessicated (Blamires and Spencer 2013; Bodie 2001; Fordham et al. 2008; Hollier 2010; Kennett et al. 1993; Micheli-Campbell et al. 2013; Riley and Litzgus 2014). The primary extinction debt for freshwater turtles in Australia is due to a lack of recruitment from excessive loss of eggs and hatchlings within nesting sites (Kuussaari et al. 2009; Van Dyke et al. 2018).

Predation of eggs from introduced species (foxes, pigs, dogs and cats) and native predators (goannas and rakali) imposes the greatest impact on population viability of threatened freshwater turtles with low fecundity, delayed maturity and aggregated nesting such as *Elusor macrurus, Elseya albagula* and *Rheodytes leukops* (Hamann et al. 2004; Limpus 2012; Limpus et al. 2011). Accordingly, improving recruitment has been given the highest priority in their conservation management. However, the efficacy of the two principal management actions to this end (predator control and nest protection), have been questioned (Campbell et al. 2020; Spencer et al. 2017). This has led to the recognition that a holistic approach including multiple management strategies that are adaptive and aimed at improving recruitment whilst addressing multiple threats is required (Mullin et al. 2020; Ocock et al. 2018).

Environmental flow (e-flow) management is one potential strategy used to achieve sustainable water resource development by mitigating impacts on aquatic species through provision of freshwater flows and levels (Arthington et al. 2018). Impacts to the timing, quality and quantity of water from river regulation affect freshwater turtles in myriad ways including movement, habitat availability and nesting ecology (Bodie 2001; Tucker 1999; Tucker et al. 2001). However, development and implementation of targeted e-flow strategies for freshwater turtles are limited (Reid and Brooks 2000). Globally, freshwater turtles have not been the primary target of e-flow strategies, meaning the benefits of watering events targeted primarily to fish, waterbirds and vegetation; have only provided observations on associated condition of turtles and their critical habitats (Gibbons and Lovich 2019; Howard et al. 2016).

The eggs of many Australian freshwater turtles do not survive inundation (Hollier 2012, 2010). Water resource development can therefore exacerbate low rates of recruitment for freshwater species including turtles through pulse and press fluctuations in water levels within, and downstream of, water storage infrastructure (Bodie 2001; Limpus et al. 2011; Marshall et al. 2015; Tucker et al. 1997). Recently, water managers have attempted to quantify and proactively manage water levels within water storage infrastructure to reduce rates of nest inundation for threatened species such as the Mary River turtle (*Elusor macrurus*) and white-throated snapping turtle (*Elseya albagula*) (Espinoza et al. 2018; McDougall et al. 2014). These management strategies operate by raising water levels during the nesting period to encourage turtles to nest higher up the bank; and then allowing levels to drop as water is used during the incubation period to minimise the impact of natural inflows approaching the wet season.

The white-throated snapping turtle (*E. albagula*) is listed as critically endangered under the Environmental Protection and Biodiversity Conservation Act (EPBC Act); and is endemic to the Fitzroy, Burnett and Mary River catchments of Queensland, Australia (Hamann et al. 2004). Anthropogenic threats affect all life-stages, however, nest predation rates approaching 100% have been recorded for *E*. *albagula* and lack of recruitment is listed as the primary extinction threat (Department of the Environment 2014). In addition, female *E*. *albagula* turtles mature at ~18 years of age, lay a single clutch per year, and have an extended incubation period (including embryo diapause) of ~6 months, increasing the risk from nest inundation (Hamann et al. 2004; Limpus et al. 2011). Priority management actions including nest protection and predator control have been implemented throughout its range, however, water level management has only been implemented within a storage on the lower Burnett River in southeast Queensland (Ben Anderson Barrage).

A specific management strategy to prevent nest inundation for *E*. *albagula* was enacted in Queensland Government legislation in 2014 (McDougall et al. 2014). This holistic strategy prescribed specific water levels for a water storage coinciding with nesting and incubation periods for this species, together with maintenance of human water security and improvement of fishway operations and estuarine flows. As part of an adaptive management approach, this study reports on a three-year monitoring program aiming to quantify annual rates of nest inundation, whilst also identifying additional economic and environmental benefits of this holistic strategy.

## Materials and Methods

### Study Area

**Fig. 1:**
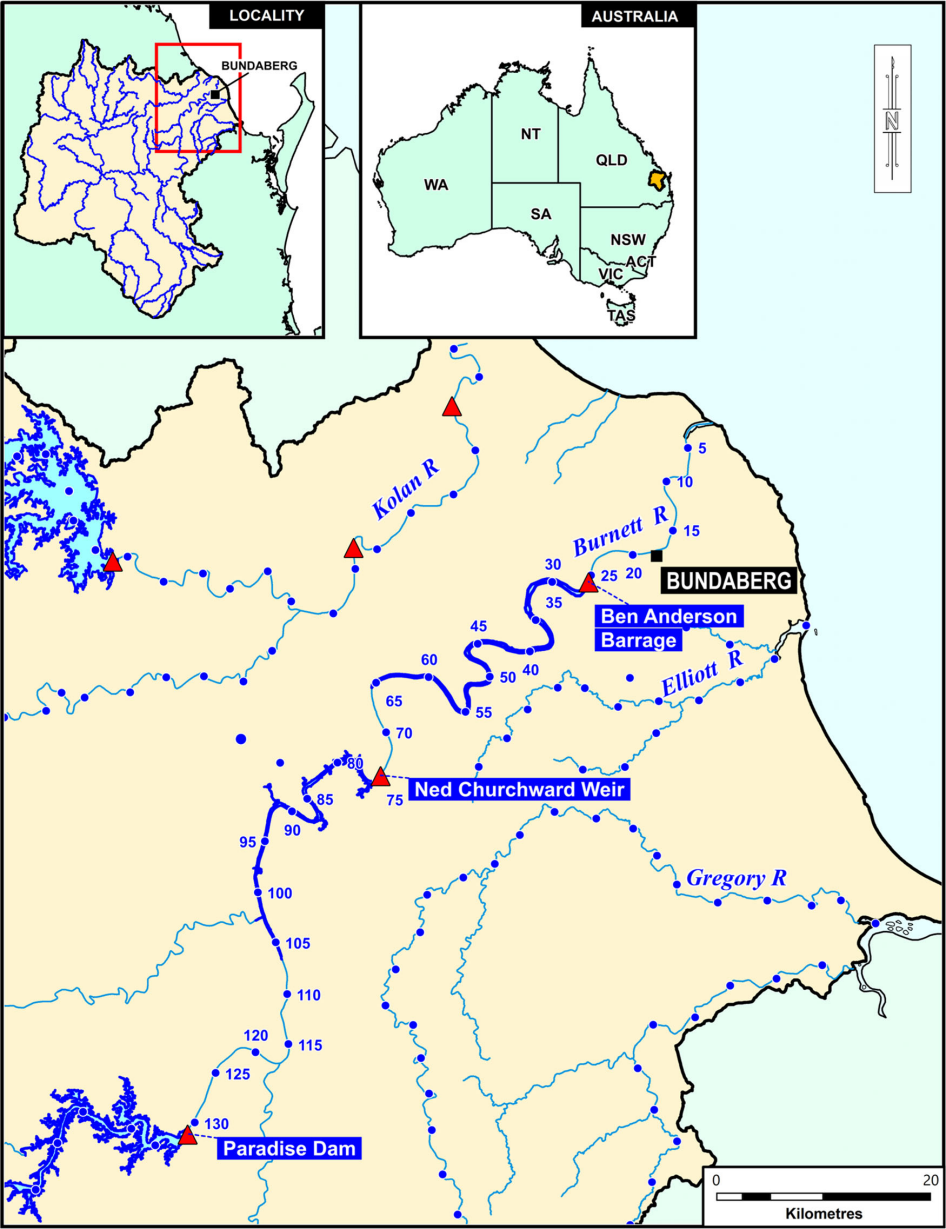
Map of general study area including the Ben Anderson Barrage and upstream water storages (Ned Churchward Weir and Paradise Dam). Blue dots = 5 km intervals Adopted Middle Thread Distance (AMTD)

The Burnett River is one of the largest in southeast Queensland, flowing for ~440 km and draining a catchment area of ~38,000 km^2^. Within the Burnett River basin, *E. albagula* nest throughout the middle to lower catchment (Hamann et al. 2004), with 90% of observed nesting occurring within the lower reaches of the Burnett River in the impounded area of the Ben Anderson Barrage (Hollier 2010) (Fig. 1).

The Ben Anderson Barrage separates freshwater and estuarine habitats at 25.9 km AMTD (Adopted Middle Thread Distance), has a full supply storage volume of 30,300 ML and impounds 40 km of river channel. The barrage is owned and operated by a Queensland Government-owned corporation (Sunwater Ltd) and managed under the ‘Water Plan (Burnett Basin) 2014’ (Queensland Government 2014). Current storage water level operating rules for the barrages and other storages within the Bundaberg Water Supply Scheme (https://www.sunwater.com.au/wp-content/uploads/Home/Schemes/Bundaberg/bundaberg-wss-operations-manual-july-2020.PDF) to reduce nest inundation of *E. albagula* are outlined in Table 1.

**Table 1:** Summary of operational rules for management of water levels in the Ben Anderson Barrage, Burnett River, as per the Water Plan (Burnett basin) 2014.

Full supply level (m AHD)	Minimum operating level (m AHD)	Fishway operating level (m AHD)	*E*. *albagula* nesting rule level (m AHD)	Period
3.97	0.0	≥2.0	≥3.0	May to July (nesting)
			≥2.2	August to April (incubation)

*AHD* Australian height datum.

### Nest Sampling

Nest surveys within the Ben Anderson Barrage impoundment were conducted during and following rain events during the *E. albagula* peak nesting season (May–July) in 2018 (*n* = 9), 2019 (*n* = 17) and 2020 (*n* = 19) as per McDougall et al. (2014). Nest identification involved scanning the riverbanks by boat and on foot looking for turtle tracks leading to nests. *E. albagula* turtles are one of only two freshwater species that nest during Autumn/Winter and have a discernible turtle track, so misidentification was highly unlikely. Eggshells from any predated clutches were used to confirm species due to the characteristic size and shape of *E. albagula* eggs (Hamann et al. 2004). *E. albagula* turtle nests were marked with a wooden stake and a unique alphanumeric code attached to identify date of nesting.

Nest characteristics were recorded, and intact clutches of eggs were moved to a caged facility to protect the eggs from predation. This work was conducted by the Department of Environment and Science (DES) under a separate project and therefore not reported on in this study. Predator effects were also not reported in this study as they were outside the main focus of nest inundation.

### Height of Nest above Water Level and Nest Inundation

Marked nests were surveyed at the end of the nesting season using global navigation satellite systems technology (Real Time Kinematics) to provide precise GPS locations and elevations (m AHD). Nest elevations for each year of monitoring were plotted onto water level data for the Ben Anderson Barrage (GS 136020B) (Sunwater 2021) for the nesting and incubation period (May–December) to assess nest inundation rates. Nest elevations were also related to water levels at time of nesting and ranked in ascending order to produce a cumulative curve for nest height above water level for 2018, 2019 and 2020. The height above water level for 20%, 50% and 80% of nests were derived using the cumulative curves. A single-factor ANOVA was also undertaken to test for any differences between nesting heights above water level for the 2007–2011 (McDougall et al. 2014) and 2018–2020 periods.

### Fishway Operation and Flows to the Estuary

Water level data for the Ben Anderson Barrage was used to determine the effectiveness of fishway operation between freshwater and estuarine environments in the lower Burnett River as the fishway is reliant on water levels in the barrage (Table 1). The fishway is operational when water levels are above 2.0 m AHD and new operational rules have been in effect since August 2014. Water level data for Woongarra pump station (GS 136020) was obtained from Sunwater for the period 01/08/2014 – 16/05/2021 and proportion of time above 2.0 m AHD calculated.

Water level data (GS 136020) was also used to assess the frequency and duration of flow events that exceeded the full supply level (overtopping events) providing freshwater flows to the estuary.

### Supply of Water for Water Allocations

Water plans are developed under the Water Act 2000 (Queensland Government 2009) to sustainably manage and allocate water resources in Queensland amongst a variety of stakeholders including irrigators, townships, Aboriginal and Torres Strait Islander peoples and the environment. Water resources include regulated and unregulated rivers, lakes and springs; overland flow and underground water. Water authorisations are required before you can take or interfere with water. A water allocation is an authority that ensures its holder access to a specific volume of water in a water supply scheme which in turn, depends on availability of water. In the Bundaberg Water Supply Scheme, along with most schemes, water allocations are assigned ‘priority’ (in this case either high or medium) – with the volume of each grouping dependent on water sharing rules and water demands in the scheme. The percentage of the allocation that each group can access at any time is calculated and announced at the start of the water year and revised throughout the water year depending on rainfall, storage volumes, time of year and other factors. ‘High’ and ‘medium’ priority announced allocation percentages were obtained from Sunwater and assessed over the 7-year period. Rainfall data was sourced from the Bureau of Meteorology (http://www.bom.gov.au/climate/data/) for station 039128.

## Results

**Fig. 2:**
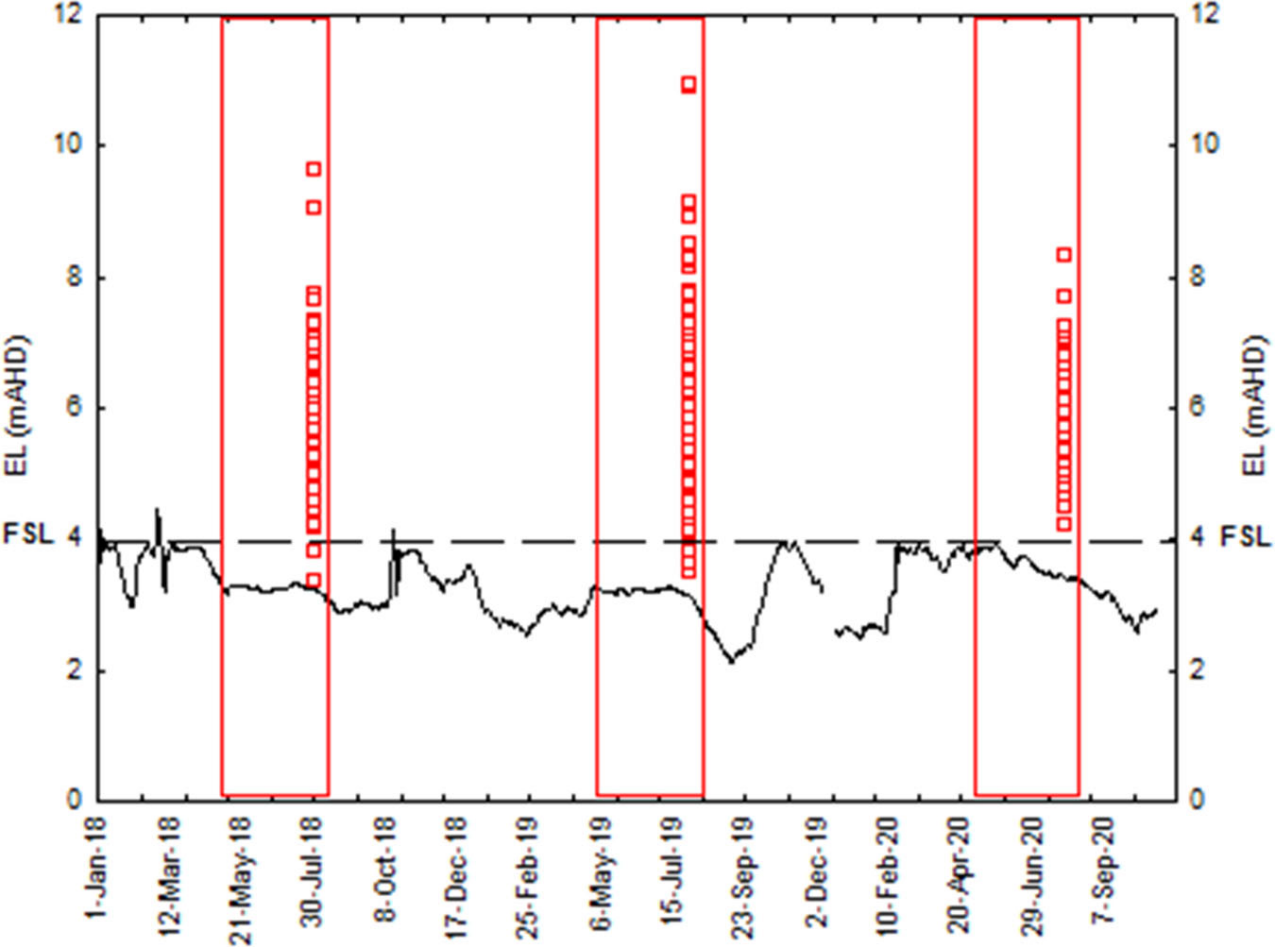
Hydrograph for the Ben Anderson Barrage (2018–2020) showing *E. albagula* nests (red squares), nesting periods (red windows) and barrage full supply level (dashed line). Note: all nests presented on the last day of the nesting season (EL = Elevation)

A total of 226 *E. albagula* turtle nests were recorded during nesting surveys in 2018 (*n* = 61), 2019 (*n* = 109) and 2020 (*n* = 56). In 2018, water levels in the Ben Anderson Barrage ranged between 3.18 m and 3.33 m AHD for the duration of the *E. albagula* turtle nesting period (May–July) (Fig. 2). During the incubation period, ~125 mm of rain between the 11th and 13th October resulted in a 1.2 m rise in water level in the barrage before overtopping into the estuary. This event inundated approximately 3% of the 61 *E. albagula* turtle nest sites recorded. In 2019, water levels in the barrage ranged between 3.13 m and 3.28 m AHD for the nesting period (Fig. 2). Emergency releases for dam safety from an upstream storage in late September and early October 2019 inundated ~11% of 109 *E. albagula* turtle nest sites recorded. In 2020, water levels in the barrage ranged between 3.39 m and 3.96 m AHD for the nesting period (Fig. 2). All 56 *E. albagula* turtle nest sites recorded were established above the 3.97 m AHD full supply level of the barrage and no significant rain events or releases from upstream storages caused any inundation of detected nests.

**Fig. 3:**
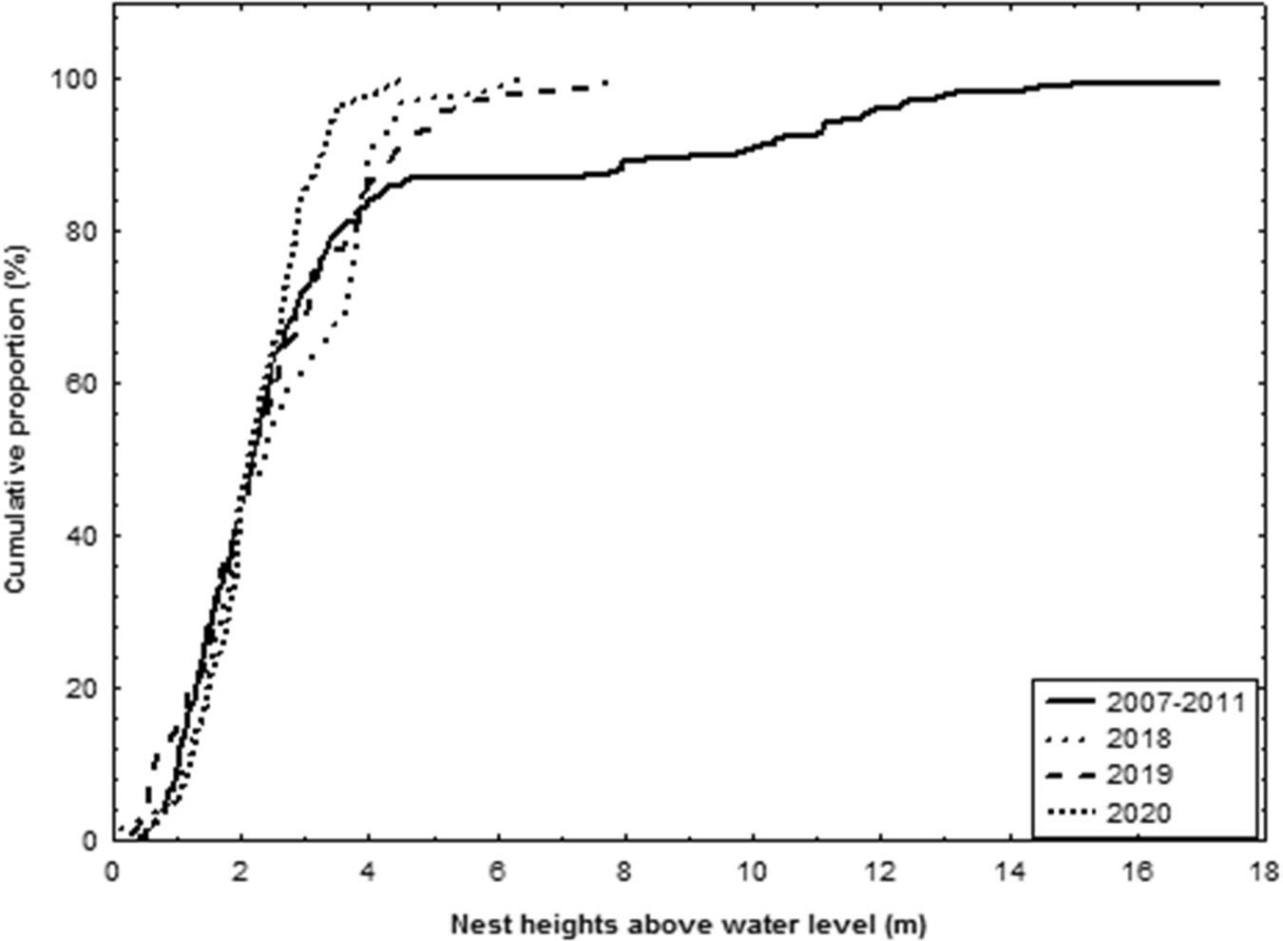
Comparison of cumulative proportion of nest heights from 2007 to 2011 (McDougall et al. 2014), 2018, 2019 and 2020

Comparison of nesting heights above water level from the 2007–2011 to the 2018–2020 period, using a single-factor ANOVA, showed no significant differences in nesting heights above water level between the two periods (*p* > 0.05) suggesting no changes in nesting behaviour in relation to water levels by female *E. albagula*. Comparison of cumulative nesting curves across the periods also showed similar 20%, 50% and 80% values, also suggesting no changes in nesting behaviour (Fig. 3).

### Fishway Operation and Flows to Estuary

**Fig. 4:**
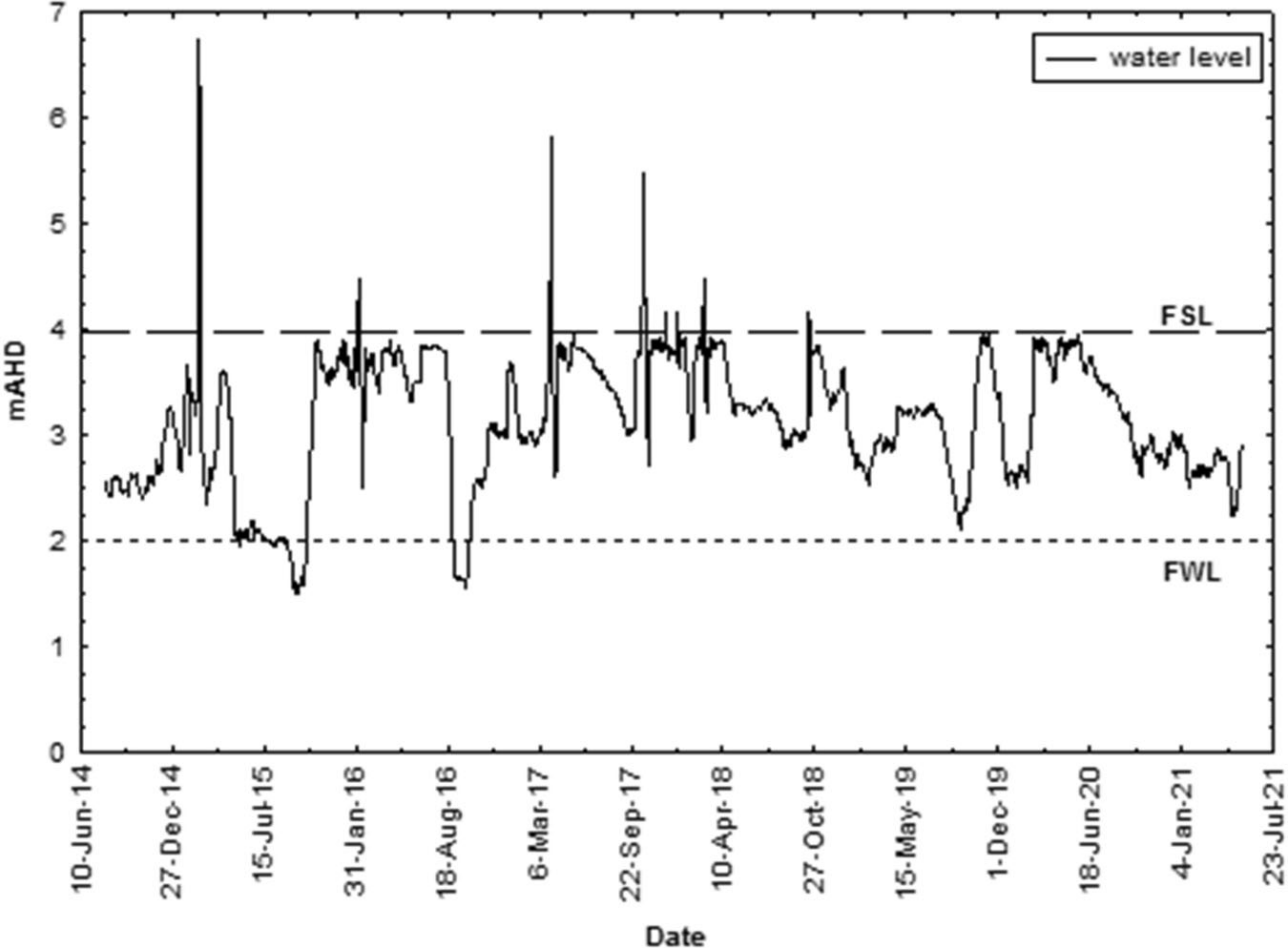
Water level data for the Ben Anderson Barrage from 2014 to 2021 indicating full supply level (FSL) and fishway level (FWL)

Fishway operation at the Ben Anderson Barrage was assessed during implementation of the Water Plan (Burnett Basin) 2014 (*n* = 2 481 days) by calculating the number of days where the water level in the barrage was above the minimum operating level for the fishway. Water levels were above EL 2.0 m AHD for >95% of the period since implementation of storage water level operational rules indicating the fishway was able to operate for this whole period (Fig. 4). Prior to 2014, the fishway was operational for only ~40% of the time using modelled water level data (1890–2009) and previous operating rules for water levels in the barrage (McDougall et al. 2014).

Freshwater flows to the Burnett River estuary were assessed from 2014 to 2021 (2481 days) during implementation of water level operations targeted towards reducing inundation of *E*. *albagula* nests. Flows overtopped the Ben Anderson Barrage on 8 occasions for a combined 34 days over the 7-year assessment period. Three of these events were during the incubation period for *E. albagula*.

### Supply of Water for Water Allocations

**Fig. 5:**
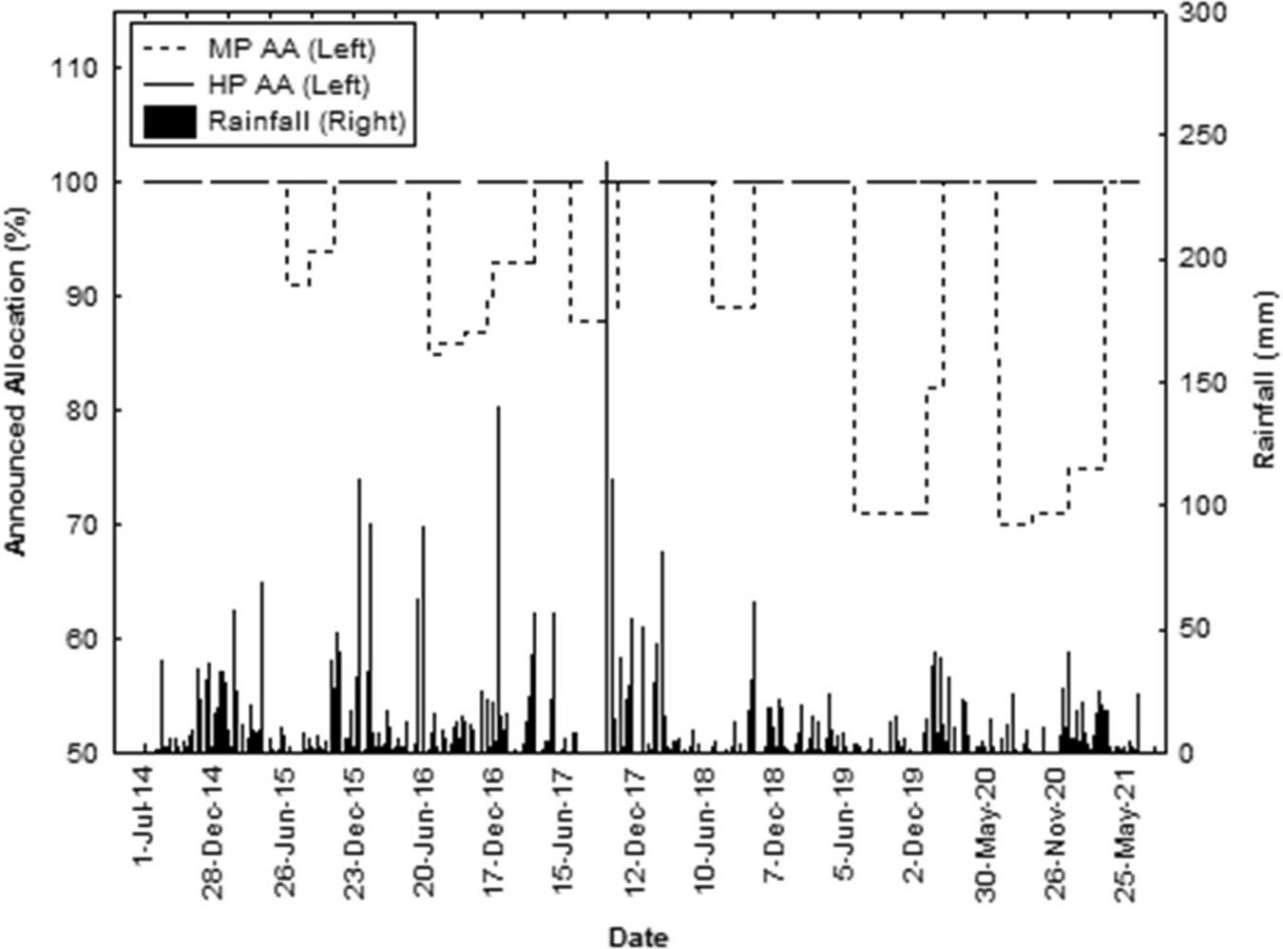
Announced allocations percentages for ‘high’ (HP AA) and ‘medium’ (MP AA) priority water allocations for the 2014–2021 period. Rainfall data for Bureau of Meteorology site 039128 also shown

Announced allocations for ‘high’ priority water allocations in the Bundaberg Water Supply Scheme were 100% for every year in the assessment period 2014 to 2021 (Fig. 5). Announced allocations for ‘medium’ priority water allocations fluctuated between 70% and 100% at the start of the water year (July) but ended at 100% in all years during the assessment period. Dry climatic conditions from mid-2018 decreased medium priority announced allocations for the Bundaberg Water Supply Scheme.

## Discussion

This study shows that active management of water levels within water storage infrastructure can reduce inundation rates of freshwater turtle nests whilst still maintaining water supply for human use and supporting additional environmental outcomes. Inundation rates for nests of the critically endangered white-throated snapping turtle were reduced through a prescribed water level regime aligned to nesting and incubation periods for this species. In addition, increased fishway operation and maintenance of freshwater flows to estuarine habitat were recorded, providing added environmental benefits. This study shows that managing water levels within water storage infrastructure can form part of a sustainable suite of strategies aiming to improve recruitment of freshwater turtles, whilst ensuring broader environmental and economic outcomes are achieved.

Freshwater turtles are in decline across the globe due to a variety of anthropogenic threats which require equally diverse management interventions (Stanford et al. 2020). Although impacts to early life-history (i.e. egg predation) are paramount, water resource development has also exacerbated these impacts through reduced connectivity, degradation and inundation of riparian nesting habitats (Norris et al. 2018). Mitigation measures such as e-flows, however, have primarily focused on fish, waterbirds and vegetation. For freshwater turtles, lack of research and monitoring into their critical life history water requirements or impacts from major instream development projects, has precluded any targeted e-flow strategies for their long term viability (Ocock et al. 2018). To the authors’ knowledge, this study represents the only implemented e-flow strategy for a freshwater turtle anywhere in the world.

Management of water levels within artificial impoundments have been included in updated definitions of e-flows (Arthington et al. 2018), and have been recently included as a recovery action in the federal recovery plan for white-throated snapping turtle specifically to reduce rates of nest inundation (National Recovery Plan for the White-throated Snapping Turtle (Elseya albagula) 2017). This recovery action was implemented due to previously estimated nest inundation rates of >20% in ~24% of years of a 119-year simulation period (1890–2009) (McDougall et al. 2014). Simulated water level data was used in this previous study in preference to measured data as the simulated data was over a longer timeframe including a more variable climatic pattern (1890–2009), compared to measured data that was only available from 1993. The measured dataset (1993–2009) also coincides with substantial changes in the management of the Bundaberg Water Supply Scheme and other schemes upstream including construction and operation of additional storages and increases in total water allocation. As such it was deemed inappropriate to compare changes in local water level management amidst a broader change in pattern of water management over a short time period.

The management of Ben Anderson Barrage water levels to minimise nest inundation has been in effect for seven years. During this time, there has been a high level of compliance with the rule, even in years where emergency releases have been made from an upstream water storage. Importantly, however, upstream water storages are critical to the effectiveness of this approach to water level management by subsidising higher water levels at the start of the nesting season and ensuring turtles nest higher up the riverbanks. Without releases from upstream storages to maintain water levels in the barrage, nest inundation rates may increase due to lower water levels during the nesting period. Whilst the rules for lowering of water levels in the barrage during the subsequent egg incubation period also aid in reducing inundation, both localised inflows and upstream flooding can affect the efficiency of the rule at this stage.

The natural distribution of *E. albagula* coincides with heavy to moderately regulated watercourses of the Fitzroy, Burnett and Mary River catchments in Queensland, Australia (Hamann et al. 2004). Nesting is believed to occur primarily in the lower reaches of all three catchments and water storage infrastructure is also present. As in the Burnett, both the Fitzroy and Mary Rivers also have tidal barrages. In addition, the Fitzroy River has multiple additional upstream water storage infrastructure; therefore, a similar approach to management of water levels could potentially be applied in this catchment if the risk of nest inundation was sufficient. In contrast, outside of its barrage, the Mary River does not have water storage infrastructure in its lower catchment to provide the capability of maintaining water levels in the barrage. Therefore, alternative management options should be considered to reduce overall threats to recruitment for this species (Campbell et al. 2020). More broadly, many other freshwater turtles in Australia, and across the globe, have similar nesting patterns and strategies to *E. albagula* but vary in the timing of nesting and level of diapause in egg development. For example, in the Mary River, the endemic *Elusor macrurus* nests in late spring or early summer and therefore has a much shorter period of risk that nests could be inundated due to a shorter incubation period (Espinoza et al. 2018).

Monitoring and assessment of implemented strategies should also be considered an essential component of adaptive management for freshwater turtles. This reduces uncertainty of the benefits of management interventions to the population viability of target species. Management programs focusing on terrestrial threats to turtles including predator control, nest protection, nesting habitat protection and headstarting have all been questioned in terms of successful species recoveries (Campbell et al. 2020; Mullin et al. 2020; Ocock et al. 2018). This has led to increased recognition of the need for more innovative, holistic and adaptive management that addresses multiple terrestrial and aquatic threats to arrest turtle declines. Furthermore, limited monitoring and assessment of e-flows and watering events in Australia have been inadequate, and have also failed to identify positive effects on target species and habitats (Albie et al. 2021; Chen et al. 2020), let alone freshwater turtle populations.

Importantly, uncertainty around assumptions relating to improved recruitment from reduced inundation, increased fish passage from increased fishway operation, and increased estuarine productivity from overtopping flows; require further investigation. Although this study has demonstrated a reduction in rate of inundation from >20% in ~24% of years of a 118-year simulation period, to an average of <5% in three consecutive years; this does not imply improved recruitment for this species. Existing threats such as egg predation may still override the benefits of reduced nest inundation, given that even low densities of foxes can decimate nesting banks (Spencer and Thompson 2005). Similarly, increased fishway operation from ~45% to ~95% of the time does not imply improved fish passage. Although the fishway may facilitate more amphidromous movements for more species throughout the year, inherent issues such as predation within fishway structures may still override the benefits (Baumgartner 2007). Finally, although freshwater flows to estuarine environments are known to facilitate movement, reproduction and growth of aquatic species (Halliday and Robins 2007); the interactive effects of timing, frequency, magnitude and duration of flow events on overall estuarine productivity are yet to be determined quantitatively. Further research and monitoring on all of these factors is warranted.

## Conclusion

Here we have shown that incorporation of ‘best available science’ into water planning, followed by effective implementation and subsequent monitoring is achievable, and a real-world example of sustainable water resource development and adaptive management. Ecological risk assessment using *E. albagula* nesting data has been used to show potential new storage operating rules could reduce rates of nest inundation for this critically endangered species (McDougall et al. 2014). Implementation and subsequent monitoring since 2014 confirmed success of the new rules, and confirmed additional benefits to fishway operation, estuarine flows and maintenance of water supply for human consumption. This process has reduced uncertainty in the efficacy of water level management for minimising nest inundation of freshwater turtles around the world. We recommend using management of water levels for reduced nest inundation within a suite of strategies in the conservation management of freshwater turtles. We also recommend a holistic and adaptive management approach where strategies targeted towards one species consider additional environmental and economic outcomes, and integrate monitoring and assessment activities to confirm win-win scenarios.
